# Cross-circulation combined with rapidly deployable venovenous bypass grafts for multiorgan biosystemic support in liver failure: experimental studies

**DOI:** 10.1097/JS9.0000000000001923

**Published:** 2024-07-02

**Authors:** Peng Liu, Lifei Yang, Qiang Lu, Yerong Qian, Aihua Shi, Xin Liu, Shasha Wei, Shujuan Fan, Yi Lv, Junxi Xiang

**Affiliations:** a Center for Regenerative and Reconstructive Medicine, Med-X Institute, The First Affiliated Hospital of Xi’an Jiaotong University; b National Local Joint Engineering Research Center for Precision Surgery and Regenerative Medicine, The First Affiliated Hospital of Xi’an Jiaotong University; c Department of Geriatric Surgery, The First Affiliated Hospital of Xi’an Jiaotong University; d Department of Hepatobiliary Surgery, The First Affiliated Hospital of Xi’an Jiaotong University; e Department of Graduate School, Xi’an Medical University; f Department of Medical Information Management, The First Affiliated Hospital of Xi’an Jiaotong University, Xi’an, People’s Republic of China

**Keywords:** coaxial electrospinning, liver failure, magnetic anastomosis, venovenous bypass

## Abstract

**Background:**

Liver failure remains a critical clinical challenge with limited treatment options. Cross-circulation, the establishment of vascular connections between individuals, has historically been explored as a potential supportive therapy but with limited success. This study investigated the feasibility of combining cross-circulation with a rapidly deployable venovenous bypass (VVB) graft for multiorgan support in a rat model of total hepatectomy, representing the most severe form of liver failure.

**Materials and methods:**

A Y-shaped VVB graft was fabricated using coaxial electrospinning of PLCL/heparin nanofibers and magnetic rings for rapid anastomosis. After total hepatectomy in rats, the VVB graft was implanted to divert blood flow. Cross-circulation was then established between anhepatic and normal host rats. Hemodynamics, biochemical parameters, blood gases, and survival were analyzed across three groups: hepatectomy with blocked vessels (block group), hepatectomy with VVB only (VVB group), and hepatectomy with VVB and cross-circulation (VVB/cross-circulation group).

**Results:**

The VVB graft exhibited suitable mechanical properties and hemocompatibility. VVB rapidly restored hemodynamic stability and mitigated abdominal congestion posthepatectomy. Cross-circulation further ameliorated liver dysfunction, metabolic derangements, and coagulation disorders in anhepatic rats, significantly prolonging survival compared to the VVB group (mean 6.56±0.58 vs. 4.05±0.51 h, *P*<0.05) and the block group (mean 1.01±0.05 h, *P*<0.05).

**Conclusion:**

Combining cross-circulation with a rapidly deployed VVB graft provided effective multiorgan biosystemic support in a rat model of total hepatectomy, substantially improving the biochemical status and survival time. This approach holds promise for novel liver failure therapies and could facilitate liver transplantation procedures.

## Introduction

Highlights

- A novel approach combining cross-circulation with a rapidly deployable venovenous bypass (VVB) graft provided effective multiorgan biosystemic support in a rat model of total hepatectomy.
- Cross-circulation significantly ameliorated liver dysfunction, metabolic derangements, and coagulation disorders in anhepatic rats, substantially prolonging survival compared to VVB alone.
- This cross-circulation and VVB graft strategy holds promise for developing new liver failure therapies and facilitating procedures like ex vivo resection and staged transplantation.

Liver failure poses significant clinical challenges due to limited treatments available for those who cannot undergo transplantation immediately^1,2^. The complexity of liver function, encompassing metabolic, synthetic, and detoxification processes, makes it difficult to manage acute liver failure effectively with current medical therapies^3–5^. The quest for alternative strategies to support patients with liver failure has led to the exploration of various bio-artificial support systems and surgical techniques.

Historically, cross-circulation^6^ was explored as a potential life-saving measure for patients with severe liver dysfunction^7,8^. In the 1960s, before the advent of artificial liver support systems and liver transplantation, cross-circulation was investigated to support patients with liver failure temporarily, and was confirmed to improve hepatic function to a certain extent^9–11^. However, the expected enhancement in overall survival was not realized^9–12^, and the approach was subsequently abandoned due to ethical concerns and technical limitations. Recent developments in immunosuppressive therapies and bioengineering have reignited interest in cross-circulation^13,14^, especially given its potential to provide comprehensive multiorgan support and internal environmental homeostasis. This resurgence is further bolstered by reports of successful utilization of cross-circulation in ex vivo organ recovery and maintenance^14–20^.

**Figure 1:**
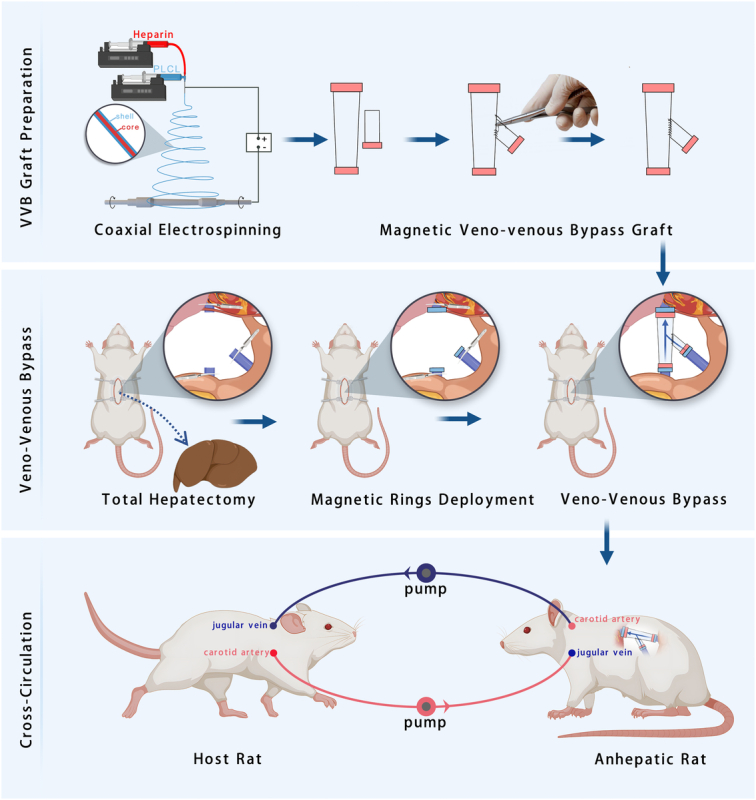
Schematic picture of the performed experiment.

In the present study, we investigated a novel approach combining cross-circulation with a rapidly deployable VVB graft to enhance multiorgan support in the treatment of liver failure (Fig. 1). The primary objective was to evaluate the efficacy of cross-circulation in supporting rats with liver failure and extending their survival. To represent the most severe form of liver failure, a rat model of total hepatectomy was employed. This model allows for a comprehensive assessment of the potential benefits of cross-circulation in the context of complete liver function loss. Furthermore, we introduced a novel technical aspect by utilizing a magnetic Y-shaped VVB graft to facilitate the rapid establishment of VVB in this model. The incorporation of this innovative graft design aimed to streamline the surgical procedure and minimize the time required to initiate VVB support. By combining cross-circulation with this rapidly deployable VVB graft, we sought to develop a comprehensive and efficient approach to managing the complex challenges associated with severe liver failure. The anticipated results of this study have the potential to provide valuable insights into the role of cross-circulation in extending survival and improving outcomes in the setting of liver failure while also highlighting the technical advancements that can facilitate its implementation.

## Materials and methods

### Materials

PLCL copolymer (15 kDa, 1 : 1 l-lactic acid to ε-caprolactone ratio, Jinan Daigang Biomaterial, China) was dissolved in hexafluoroisopropanol (Macklin, China) to prepare a 200 mg/ml shell solution. Heparin (Runjie Chemical Reagent, China) was dissolved in normal saline to prepare a 100 mg/ml core solution. FITC-DEX (20 kDa, Sigma, USA) was dissolved in distilled water to a concentration of 1 mg/ml. NdFeB magnetic rings (MR) were acquired from Xi’an Magnat Medical Technical and Jiujiu High-Tech Magnetic Materials. For suprahepatic vena cava (SHVC) anastomosis, a runway-shaped MR-SHVC measures 8 mm by 5.5 mm with a 1 mm thickness. The infrahepatic vena cava (IHVC) anastomosis ring (MR-IHVC) has a 5.8 mm outer diameter, 4.0 mm inner diameter, and is 1.5 mm thick. For portal vein (PV) anastomosis, the ring (MR-PV) features a 4.9 mm outer diameter, 3.3 mm inner diameter, and 1.5 mm thickness.

### Electrospinning and venovenous bypass grafts preparation

Electrospun collectors were created using a 3D printer (Anycubic, China), including a working collector and an auxiliary collector. Electrospinning was performed with an ET-2535H electrospinner (Yongkang Leye, China). For coaxial electrospinning, PLCL shell solution was fed at 2 ml/h through a 17 G outer capillary and heparin core solution at 0.2 ml/h through a 22 G inner capillary. The setup was maintained at 18 kV and a 15 cm distance. PLCL nanofiber electrospinning used a 22 G needle with a voltage of 9–10 kV. Other parameters matched those in coaxial electrospinning.

**Figure 2:**
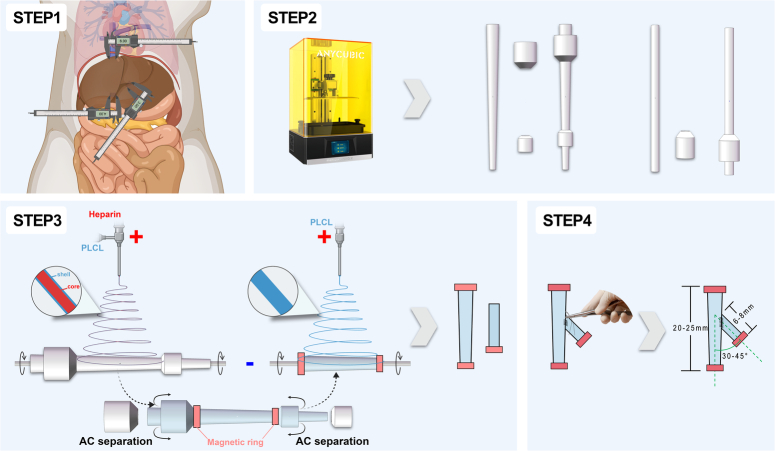
Workflow of venovenous bypass graft preparation. Step 1: measuring the diameter of the liver’s blood vessels. Step 2: fabricating WCs and ACs using a 3D printer. Step 3: creating a bilayer for VVB graft using electrospinning. Step 4: suturing the branch of the VVB graft using end-to-side anastomosis. AC, auxiliary collector; VVB, venovenous bypass; WC, working collector.

The fabrication of the VVB grafts has been previously described^21^ with minor modifications (Fig. 2). The VVB grafts’ inner layers were produced using coaxial electrospinning, with the collector rotating at 75 rpm. After 20 min, the auxiliary collectors were removed, MRs were inserted, and the membrane was inverted to cover the rings. The rotation speed was then reduced to 50 rpm for PLCL electrospinning to encase the MRs. The graft’s branch was secured with 8-0 nylon suture via end-to-side anastomosis. Electrospun membranes were vacuum-dried overnight for solvent removal and sterilized by cobalt-60 irradiation.

### Characterization of nanofibers and venovenous bypass graft

#### Micromorphology of electrospun nanofibers

Transmission electron microscopy (H-7650, Hitachi, Japan) was used to verify the core-shell structures within the electrospun nanofibers. Scanning electron microscopy ( TM-1000, Hitachi, Japan) was used to observe the electrospun fibers and cross-sections of the vascular bypass grafts. The coaxial structure was visualized by adding FITC-DEX (0.1 mg/ml) to the core solution, followed by fluorescence microscopy imaging.

#### Fourier transform infrared spectroscopy

Fourier-transform infrared spectrometry (Nicolet iS50, Thermo, USA) was used for analyzing PLCL fibers, PLCL/heparin fibers, and heparin, employing transmission mode over a 4000–400 cm^−1^ range with a 4 cm^−1^ resolution and 64 scans.

#### In-vitro heparin release

For in-vitro heparin release detection, 300 mg of the sample was soaked in 10 ml of PBS at 37°C. Periodically, 1 ml of the medium was refreshed with new PBS. Triplicate assays of each sample were conducted using a quantitative heparin assay kit (G-Clone, China).

#### Hemocompatibility analysis

To assess hemocompatibility, platelet adhesion, hemolysis, and clotting time were evaluated. For platelet adhesion, a 1 cm^2^ sample was immersed in platelet-rich plasma from anticoagulated rabbit blood and incubated at 37°C for 1 h. Scanning electron microscopy was employed to observe platelet adhesion morphology and quantity. In the hemolysis test, a 1 cm^2^ sample was incubated in saline at 37°C for 30 min, followed by the addition of diluted rabbit blood and further incubation for 1 h. The absorbance of the supernatant was measured at 540 nm postcentrifugation. Clotting time was assessed via prothrombin time (PT), activated partial thromboplastin time (APTT), and plasma recalcification time (PRT) measurements. Samples (1 cm^2^) were incubated with 2 ml of platelet-poor plasma at 37°C for 15 min for PT and APTT, and 10 min for PRT. An automated analyzer was utilized for PT and APTT measurements. For PRT, clot formation time was recorded after adding 0.025 M calcium chloride postincubation.

#### Mechanical properties

The mechanical properties and magnetic force of the VVB grafts were evaluated using a universal testing machine (CMT8502, New Sans Test, China). The samples were subjected to tensile testing at a constant loading rate of 10 mm/min until failure. The burst pressure was measured using a biological signal acquisition and analysis system (BL-420F, Taimeng, China).

### Cross-circulation experiment

#### Animal grouping

Forty-five male Sprague-Dawley rats (250–300 g) were randomly divided into three groups (*n*=15 each): block, VVB, and VVB/cross-circulation groups. The allocation was performed using a computer-based random order generator. Rats in the block group underwent PV and inferior vena cava (IVC) block after total hepatectomy. Rats in the VVB group underwent VVB after total hepatectomy. The VVB/cross-circulation group underwent VVB with simultaneous cross-circulation after total hepatectomy. The rest of the management procedure will be consistent between the three groups. All rats were managed under normal husbandry conditions. Animals with unsuccessful procedures at any stage were removed from the study. Animal experiments were approved by the Institutional Animal Care and Use Committee of Xi'an Jiaotong University. This work has been reported in accordance with the ARRIVE guidelines^22^.

#### Total hepatectomy

Before surgery, rats were fasted for 12 h and deprived of water for 6 h. Continuous anesthesia with isoflurane was maintained during surgery. A ventral midline incision was made. Initially, the common bile duct and hepatic artery were ligated and excised. Following that, the ligaments surrounding the liver were sequentially dissected to detach the liver completely. Vascular clamps were used to block the PV and IHVC. Normal saline was injected into the PV to clear the blood from the liver. The SHVC was blocked using a Satinsky clamp, and the PV and IHVC were then cut off. Finally, each liver lobe was resected, maintaining a distance of 5 mm from the root.

#### Establishing an anhepatic rat model via venovenous bypass graft implantation

The method described earlier was used with slight modifications^21^. For the SHVC, a MR-SHVC was first inserted at the end of SHVC, with its vessel wall inverted to cover the ring’s edge. The lumen was filled with saline before magnetically connecting the VVB graft for complete anastomosis. The IHVC and PV underwent similar anastomosis processes. Clamps on the SHVC, IHVC, and PV were then removed. The abdominal cavity was irrigated with 37°C saline, followed by incision suturing. Postoperatively, flurbiprofen axetil (5 mg/kg) was administered intraperitoneally for analgesia.

#### Cannulation

Initially, a midline incision in the neck was made, followed by clamping of the carotid artery using microscopic forceps and ligation distally with a 6-0 suture. Subsequently, the proximal end of the artery was clamped with a vascular clip. A small incision was then made distal to the ligation, and a carotid artery cannula was inserted approximately 1 cm into the artery and secured using a 6-0 suture. The jugular vein was cannulated by using a similar approach. Subsequently, the cannula was tunneled subcutaneously to the back of the neck and fixed using a 3D-printed vascular access port.

#### Cross-circulation

To start the procedure, the carotid artery and jugular vein cannulas of anhepatic and host rats were connected to a double-channel peristaltic pump using 18 G stainless steel tubes. The pump speed was initially set to 1 ml/min and gradually increased to 4 ml/min over the first 15 min. Blood samples were collected at regular intervals for biochemical and blood gas analysis.

#### Hemodynamic parameters

Hemodynamic parameters were continuously monitored using a biological signal acquisition and analysis system (BL-420F, Taimeng, China). The parameters included the blood pressure, heart rate, PV pressure, and pressure in the VVB graft.

#### Biochemical and blood gas analysis

Coagulation activity was evaluated using an automated coagulation analyzer (RAC-030, Rayto Life Science, China). Liver function and blood ammonia levels were assessed using kits as per the Nanjing Jiancheng Institute guidelines. Blood gas analysis was performed using an ABL 800 FLEX analyzer (Radiometer, Denmark).

### Statistical analysis

Raw data were analyzed in a blinded way. Data analysis was conducted using the SPSS software (22.0, SPSS Inc., USA). Quantitative data are presented as the means±SD. Multiple‐group comparisons were performed by analysis of variance. Differences between the two groups were compared by *t* test. Statistical significance was set at *P* value <0.05, with levels indicated as *P value <0.05, **P value <0.01.

## Results

### Characterization of electrospinning nanofibers

**Figure 3:**
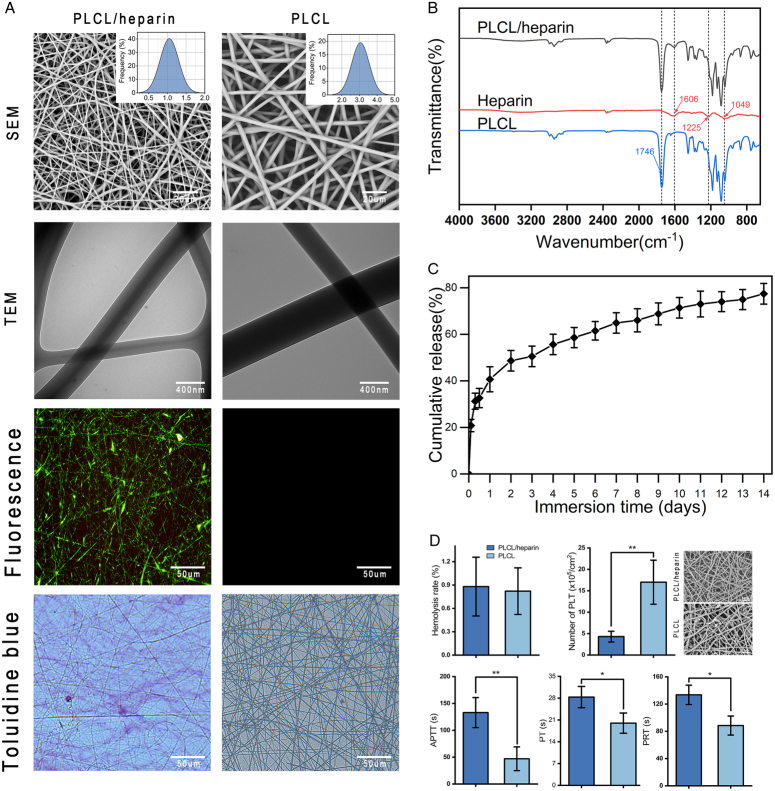
Characterization of electrospinning nanofibers. (A) SEM, TEM, fluorescence images and toluidine blue staining of PLCL/heparin and PLCL nanofibers. (B) FTIR spectra of nanofibers. (C) Heparin release in vitro. (D) Hemocompatibility. FTIR, Fourier-transform infrared spectrometry; SEM, scanning electron microscopy; TEM, transmission electron microscopy.

The characterization of electrospinning nanofibers has been previously described^21,23^. Briefly, both types of nanofibers exhibited smooth and uniform morphology without defects. The average diameters of the PLCL/heparin and PLCL nanofibers were 1.05±0.23 and 3.06±0.49 μm, respectively (Fig. 3A). Transmission electron microscopy images visually revealed the core-shell structures of the PLCL/heparin nanofibers, showing a clear contrast between the core and shell. The core diameter accounted for 32.7±4.8% of the entire fiber, and fluorescent dyes added to the core solution demonstrated a uniform distribution along almost all nanofibers.

The Fourier-transform infrared spectrometry spectra (Fig. 3B) of heparin, PLCL, and PLCL/heparin nanofibers displayed overlapping characteristic peaks (1746 and 1606 cm^-1^), indicating physical encapsulation of heparin.

Heparin release from two nanofiber types was assessed using toluidine blue, which was selected for its colorimetric reaction with heparin. PLCL nanofibers retained the original blue color of toluidine blue, indicating no reaction (Fig. 3A), whereas PLCL/heparin nanofibers caused the color to shift to purple, signaling the release of heparin. In-vitro experiments quantified heparin release from PLCL/heparin nanofibers, highlighting a two-phase release pattern: an initial burst within the first 2 days, then a sustained release (Fig. 3C). On day 1, about 40.7% of heparin was released, with a consistent release thereafter. By day 14, around 77.4% of the total heparin was released.

In terms of hemocompatibility, the PLCL/heparin nanofibers demonstrated improved performance compared the PLCL nanofibers, as evidenced by significantly lower platelet adhesion and aggregation (Fig. 3D). The hemolysis rates for both types of nanofibers were below 2%, meeting the ASTM-F756-17 standards. Additionally, the results indicated that PT, APTT, and PRT were significantly prolonged in PLCL/heparin nanofibers (Fig. 3D).

### Characterization of Y-shaped venovenous bypass graft

**Figure 4:**
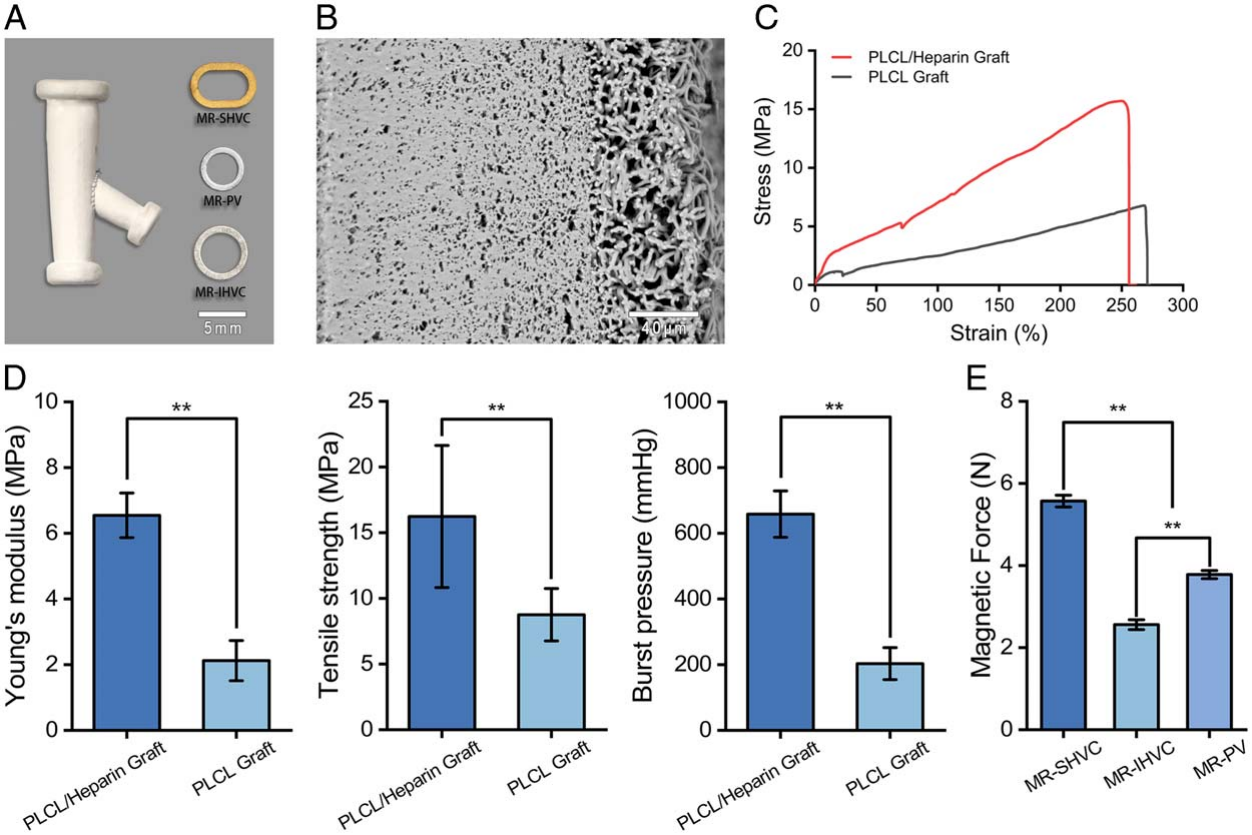
Characterization of Y-shaped VVB Graft. (A) Gross appearance of the Y-shaped VVB graft and MRs. (B) The cross-sectional SEM image of the graft. (C) Stress-strain curves. (D) Young’s modulus, tensile strength, and burst pressure of the grafts. (E) The magnetic force between the runway-shaped and ring-shaped MRs. IHVC, infrahepatic vena cava; MR, magnetic ring; PV, portal vein; SHVC, suprahepatic vena cava; VVB, venovenous bypass.

The distance between the diaphragm and superior renal vein edge in 250–300 g male Sprague-Dawley rats was 18.32±6.61 mm. The angle between the PV and the IVC was 34.24±8.68°. Using these anatomical data, we determined the parameters for each component of the Y-shaped VVB graft (Figs 2, 4A). The trunk length was 20–25 mm. The angle between the PV branch and trunk was 30–45°. PV branch length was 6–8 mm (Fig. 2).

The mechanical properties of the grafts were characterized by Young’s modulus, tensile strength, and burst pressure, as shown in Figure 4C–D. The Young’s modulus (6.55±0.68 vs. 2.12±0.61 MPa, *P*<0.01), tensile strength (16.23±5.41 vs. 8.75±1.99 MPa, *P*<0.01) and burst pressure (658.31±70.68 vs. 203.12±48.67 mmHg) of the grafts made of PLCL/heparin nanofibers were much larger than those of the grafts made of pure PLCL nanofibers. The magnetic forces of MR-SHVC, MR-IHVC, and MR-PV were 5.569±0.143, 2.56±0.12, and 3.78±0.1 N, respectively. Hence, the mechanical properties of the grafts are sufficient for VVB.

### Effect of venovenous bypass on abdominal organs and hemodynamics

**Figure 5:**
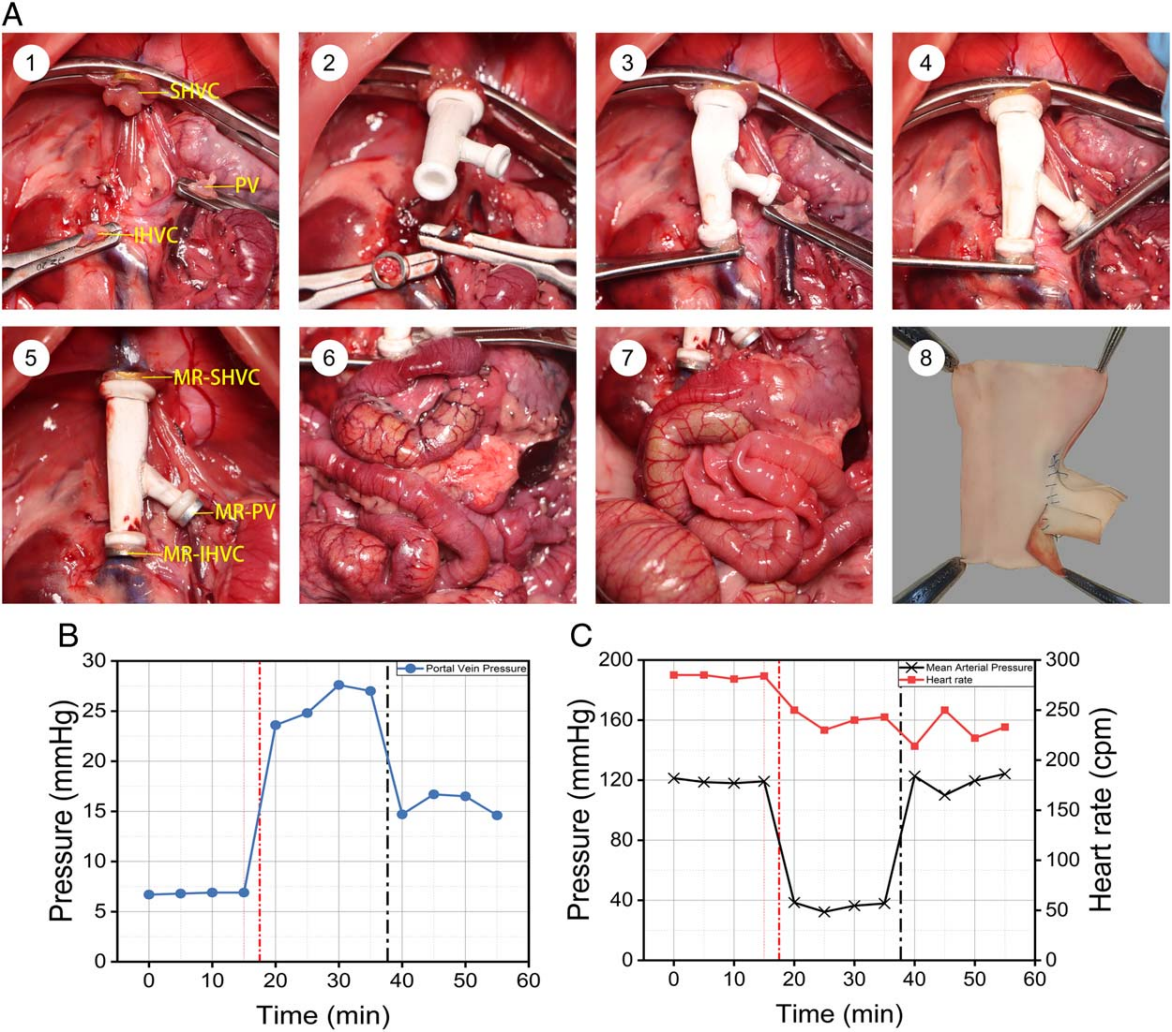
Hemodynamic and survival assessment. (A) Surgical process of VVB graft implantation. (1) The entire liver was excised, and a runway-shaped MR-SHVC was installed; (2) the VVB graft was magnetically attached to the MR-SHVC, and the MR-IHVC was installed; (3) the IHVC anastomosis was completed; (4) the PV anastomosis was completed; (5) three vascular clamps were removed; (6) during the block phase, surface vascular congestion and dilation of abdominal organs occur. The gastrointestinal tract appeared purplish-black; (7) during VVB, the abdominal organ congestion is resolved, and the gastrointestinal tract color returns to bright red; (8) after VVB, the VVB graft is dissected, revealing no thrombosis inside. (B) Changes in portal vein pressure before and after VVB. (C) Hemodynamic changes before and after VVB. IHVC, infrahepatic vena cava; MR, magnetic ring; PV, portal vein; SHVC, suprahepatic vena cava; VVB, venovenous bypass.

VVB diverts blood from the portal venous system into systemic circulation, alleviating congestion in abdominal organs and reducing ischemia-reperfusion injury. Figure 5A illustrates the changes in vascular state. Pre-bypass images showed marked tortuosity and dilation of abdominal vasculature with severe congestion. Post-bypass, organs regained their normal color, indicating congestion resolution. Figure 5B shows a decrease in PV pressure from 28 to 16 mmHg after the bypass. The mean vascular block time was 13.4±4.5 min. No intra-abdominal hemorrhage, anastomotic leakage, or thrombosis within the graft was observed after VVB (Fig. 5A).

Block of the IVC and PV causes congestion of the abdominal organs and reduced cardiac output, leading to hypotension and bradycardia. Figure 5C shows the pre-bypass and post-bypass alterations in mean artery pressure and heart rate. Following the block, there was a precipitous decline in mean artery pressure from 120 to 40 mmHg and a concomitant reduction in heart rate. Conversely, VVB restored blood pressure to pre-block levels.

### Optimization and outcomes of cross-circulation

**Figure 6:**
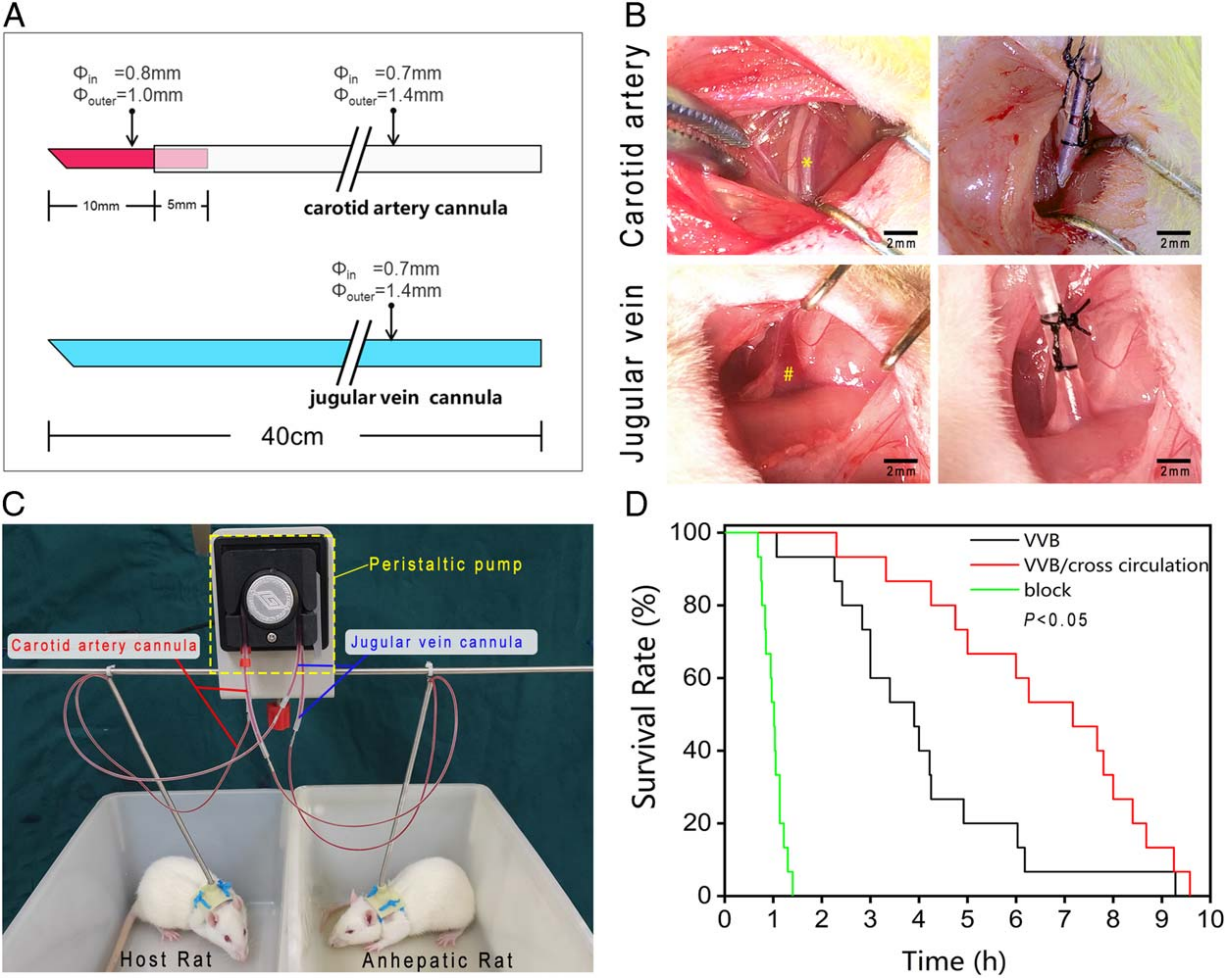
Optimization and outcomes of cross-circulation. (A) Dimensions of the cannulas. (B) Placement of cannulas in the carotid artery (*) and jugular veins (#) for cross-circulation. (C) The host and anhepatic rat during cross-circulation. (D) Survival profile of the block, VVB, and VVB/cross-circulation groups. VVB, venovenous bypass.

To minimize blood volume in the cross-circulation system, silicone tubes with small inner diameters were used for cannulation (Fig. 6A). The carotid artery cannula consisted of a 15 mm long epidural catheter (inner diameter: 0.8 mm, outer diameter: 1.0 mm) connected to a silicone tube (inner diameter: 0.7 mm, outer diameter: 1.4 mm). The jugular venous cannula was a 40 cm long silicone tube with an inner diameter of 0.7 mm and an outer diameter of 1.4 mm. The blood volume in the cannulas was 615.75 μl, representing 3% of the total blood volume (20 ml) of the two rats. Blood exchange between the two rats occurred every 10 min, with no thrombus formation observed in the cannulas during cross-circulation.

One end of the cannulas was inserted and secured into the common carotid artery and jugular vein of the rats, respectively (Fig. 6B), while the other end was connected to a dual-channel peristaltic pump (Fig. 6C). Upon initiating cross-circulation, the anhepatic rats in the VVB/cross-circulation group maintained normal respiration and activity, exhibiting behaviors such as exploring, eating, and drinking. The average survival time for these rats was 6.56±0.58 h, with a median survival time of 7.17 h, significantly longer than that of rats in the VVB or block group. The host rats showed no acute immune rejection signs, like dark urine, respiratory issues, or skin rashes, during cross-circulation. Furthermore, they survived long-term postdisconnection from the system.

Initially, the anhepatic rats in the VVB group exhibited stable respiration and engaged in spontaneous activities. However, their condition deteriorated over time, as evidenced by diminished spontaneous activity, lethargy, aversion to water, and tremors in some instances, indicative of liver failure and hepatic encephalopathy. They presented an average survival time of 4.05±0.51 h and a median survival time of 3.9 h (Fig. 6D).

In contrast, the anhepatic rats in the block group did not regain consciousness, and these rats had an average survival time of 1.01±0.05 h and a median survival time of 1.02 h.

### Biochemical and blood gas analysis

**Figure 7:**
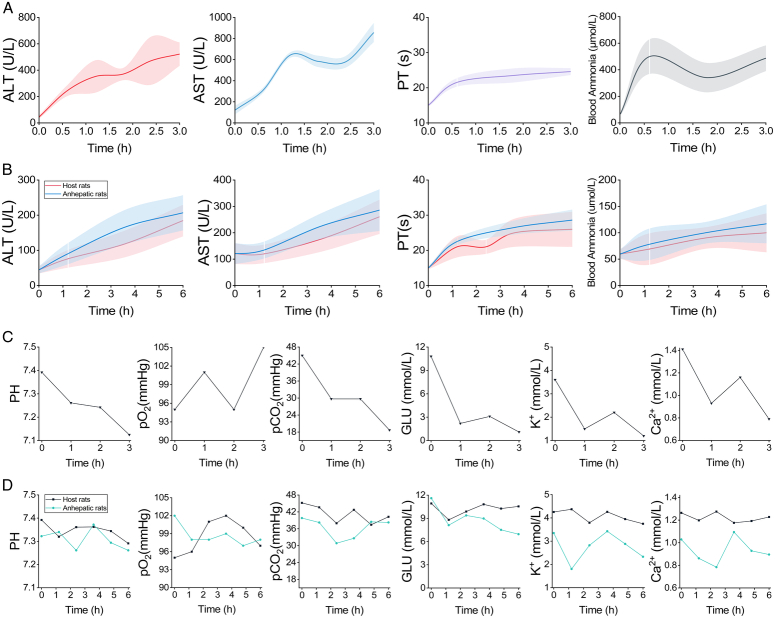
Blood–gas and biochemical analysis. (A) Biochemical analysis of the VVB group. (B) Biochemical analysis of the VVB/cross-circulation group. (C) Blood gas analysis of the VVB group. (D) Blood gas analysis of the VVB/cross-circulation group. VVB, venovenous bypass.

Liver function and blood ammonia levels were assessed to confirm changes in general conditions. Figure 7A illustrates the increases in alanine aminotransferase (ALT), aspartate aminotransferase (AST), and blood ammonia levels in the anhepatic rats of the VVB group. ALT levels increased tenfold, rising from a baseline of 44.34±10.00 to 521.94±87.31 U/l 3 h later, while AST levels experienced a sixfold increase from 120.60±39.25 to 856.71±88.27 U/l within the same period. The PT remained relatively stable at around 20 s. Blood ammonia levels escalated from baseline to 99.96±36.89 μmol/l after surgery, indicating severe liver dysfunction and hepatic encephalopathy.

In contrast, the VVB/cross-circulation group showed a significant difference. After 6 h, the ALT of the host rats increased to 184.62±45.10 U/l, which was about three times the normal value (Fig. 7B). Similarly, the ALT and AST levels in the anhepatic rats rose to 206.75±50.45 and 285.84±79.23 U/l, respectively, which were three and two times higher than the normal values. The PT value and blood ammonia levels also increased as the survival time extended. The PT value in the anhepatic rats reached 28.61±3.26 s after 6 h, compared to the preoperative normal value, while the PT value in the host rats was 26.5±5.12 s. The blood ammonia levels in both rats also increased from the preoperative normal value to 99.96±36.89 and 116.96±36.89 μmol/l, respectively. These values were significantly lower than those of anhepatic rats in the VVB group (*P*<0.05).

The anhepatic rats also exhibited abnormalities in blood gas, glucose, and electrolyte levels. Figure 7C displays outcomes for anhepatic rats in the VVB group. Post-VVB, metabolic acidosis, respiratory alkalosis, hypocalcemia, hypokalemia, and hypoglycemia were observed, worsening over time. Figure 7D illustrates the typical blood gas analysis results in host and anhepatic rats of VVB/cross-circulation group. Despite considerable variability in PO_2_, it remained within the normal range, indicating the respiratory function remained essentially unchanged. Cross-circulation also effectively corrected glucose and electrolyte imbalance in anhepatic rats, resulting in a significant enhancement in conditions of hypocalcemia, hypokalemia, and even hypoglycemia. Although the blood gas analysis parameters in the anhepatic rats did not completely normalize, they exhibited a substantial improvement.

## Discussion

Cross-circulation has been studied and confirmed to improve hepatic function, yet challenges related to survival improvement and technical limitations persist^9–12^. The present study demonstrates that cross-circulation, combined with a rapidly deployable VVB graft, effectively mitigates the severe consequences of liver failure in a rat model of total hepatectomy. Cross-circulation significantly improved biochemical parameters and survival compared to VVB alone, highlighting its potential as an effective treatment method for managing complete liver function loss. The introduction of a novel magnetic Y-shaped VVB graft facilitated rapid VVB establishment, showcasing the technical innovation that can streamline the implementation of this combined therapy. Our results provide compelling evidence for the benefits of cross-circulation, supported by rapidly deployable VVB, in the management of severe liver failure.

The key finding of this study is that cross-circulation, as a multiorgan biosystemic support approach, can mitigate liver dysfunction, blood gas abnormalities, electrolyte imbalances, and coagulation disorders in anhepatic rats, which can be considered as a model of most severe liver failure. Besides, improved survival was achieved in the anhepatic rats. Historically, cross-circulation has been explored since the 1960s for liver failure management^7–11^, predating artificial liver and transplant solutions. Recent advances in immunosuppressants and humanized animal models have reignited interest in this technique. Brandon A Guenthart *et al*.^18,20,24–26^ have documented its application for supporting and recovering human livers^16,20^ and lungs^15,25^. Our results further indicate that cross-circulation, as a multiorgan biosystemic support method, potentially aids in the physiological regulation and management of liver failure. This effectiveness is mainly due to its ability to replicate critical physiological processes necessary for sustaining life, encompassing multiorgan synthesis, neurohormonal signaling, and intricate interorgan communication^13,14,18^. Current artificial organ systems lack these capabilities.

A notable difference from previous studies is the employment of a total hepatectomy animal model. Previous efforts with cross-circulation in treating liver failure demonstrated minimal improvement in symptoms and biochemical markers without significantly prolonging survival^7,9–11^. In contrast, this study demonstrated enhanced outcomes with cross-circulation attributed to the underlying etiology. Specifically, many liver failure patients experience toxic liver syndrome, induced by conditions such as fulminant hepatic failure, severe liver trauma, or primary graft nonfunction, characterized by liver necrosis, shock, renal failure, and respiratory failure, leading to severe systemic inflammatory response^27–31^. Total hepatectomy can benefit these patients by removing necrotic or ischemic-hypoxic liver tissue, which has irreversibly lost its normal function and releases harmful substances that aggravate systemic conditions. Thus, total hepatectomy can stabilize metabolism and facilitate other resuscitation measures.

However, total hepatectomy inevitably induces severe hemodynamic disturbances, affecting multiple organs. A significant innovation in this research is the use of a magnetic Y-shaped VVB graft fabricated through coaxial electrospinning and magnetic anastomosis techniques to address this. This graft enables rapid bypass deployment after total hepatectomy, stabilizing blood flow dynamics and metabolism and facilitating rapid liver transplantation. Our previous research confirmed the reliability and superiority of similar magnetic artificial blood vessels^23^. Magnetic anastomosis speeds up the bypass procedure compared to traditional suturing, eliminating the need for extra incisions, catheterizations, or special equipment. Combined with magnetic liver transplantation^32,33^, it shortens PV block time, which is associated with better outcomes in liver surgery. Additionally, the controlled release of heparin prevents anticoagulation without additional anticoagulants, reducing bleeding risks.

While cross-circulation did extend the survival time of anhepatic rats, it was insufficient for their long-term survival due to several factors. First, total hepatectomy induced severe trauma that exceeded the rats’ tolerance threshold. Second, the resistance of the Y-shaped VVB graft was lower than that of the liver, leading to increased venous return to the heart, which could potentially compromise cardiac function^34^. Lastly, despite cross-circulation, metabolic byproducts and toxins could directly enter the systemic circulation via the graft, impacting vital organs such as the heart, brain, and kidney^28^. Therefore, future research will focus on the selection of animal models and further optimization of the graft.

The results of this study suggest potential clinical applications in two-stage liver transplantation and *ex vivo* liver resection. For instance, in patients suffering from fulminant hepatic failure due to toxic syndromes or massive hemorrhage, and who lack suitable liver donors^27–29,31^, the damaged liver can be excised, and rapid VVB can be initiated. During this period, the patient can be maintained through xenogeneic cross-circulation, which serves as a multiorgan biosystem support until a suitable liver becomes available. Furthermore, the novel magnetic anastomosis technology developed in this study greatly simplifies and expedites the surgical procedures involved. By utilizing magnetic anastomosis technology, VVB^21^ and liver transplantation^32^ can be performed swiftly, making the process as straightforward as assembling Lego pieces. Similarly, this approach can be applied in *ex vivo* liver resection for patients with extensive liver tumors or other space-occupying lesions that are considered unresectable by conventional means^35,36^. In such cases, the diseased liver can be removed, and the patient can be maintained on VVB and cross-circulation while the liver undergoes *ex vivo* resection. The magnetic anastomosis technology can then be employed to reconnect the liver’s vascular and biliary structures before reimplantation into the patient. This innovative technique not only expands the boundaries of liver surgery but also provides a safer and more controlled environment for complex liver resections, potentially improving surgical outcomes and reducing complications.

In this study, we employed allogeneic rats for cross-circulation as a preliminary confirmation experiment. However, the ultimate goal is to establish xenogeneic cross-circulation. Xenogeneic cross-circulation is considered more feasible than xenogeneic organ transplantation for several reasons^15,16^. First, cross-circulation allows for the continuous exchange of blood and its components between the donor and recipient, potentially reducing the immunological challenges associated with solid organ transplantation^15,16^. Second, the donor animal can remain alive during the cross-circulation process, allowing for the continuous supply of essential factors and the removal of waste products. This dynamic exchange may help to maintain the homeostasis of the recipient’s internal environment. Third, cross-circulation can be performed as a temporary intervention, providing a bridge to support the recipient while waiting for a suitable allogeneic organ or allowing time for the recipient’s own organ to recover. In contrast, xenogeneic organ transplantation requires the immediate and permanent replacement of the recipient’s organ with a xenogeneic graft, which poses significant immunological and physiological challenges. Over the past decade, considerable progress in biotechnology and genetic engineering has facilitated the development of genetically engineered pigs specifically for human organ transplantation^37–40^. Despite these advances, the persistent risk of chronic immune reactions to xenoantigens continues to pose a significant challenge, compromising both the longevity and functionality of the transplanted organs^41–43^. Therefore, the temporary nature of xenogeneic cross-circulation may provide a more feasible approach to supporting patients with end-stage organ failure while minimizing the long-term immunological complications associated with permanent xenogeneic organ transplantation.

While the current study demonstrates the potential of cross-circulation, it is important to acknowledge the inherent differences between rodents and humans. To ensure the safety and efficacy of this approach, extensive validation in large animal models is necessary. Moreover, several ethical and clinical concerns surrounding cross-circulation must be addressed before its application in human patients. Firstly, the use of genetically modified humanized^44^ or immunodeficient swine (such as antibody-deficient or severe combined immunodeficient swine^45,46^) for xeno-support may be the most crucial immunomodulatory strategy. This approach could significantly reduce the risk of immune rejection and enhance the compatibility between the xenogeneic support system and the human recipient. Secondly, additional pharmacologic^47,48^ and mechanical strategies^49^ should be employed to diminish the immunologic reaction against xenoantigens. These may include T cell-depleting therapies, IL-2 receptor antibodies, and leukocyte filters, which can help to reduce the immunogenicity of the xenogeneic support system^14,17^. Future studies should focus on optimizing techniques to minimize immunologic responses during cross-circulation, paving the way for safe and effective clinical applications.

The current study had several limitations. First, we used a small animal model, which exhibited low tolerance to the anhepatic state and extensive surgical procedures. Future investigations should employ large animal models such as pigs. In addition, the potential application of xenogeneic cross-circulation needs to be validated using humanized animals through immunosuppressive drugs or gene editing.

## Conclusion

This study demonstrates the feasibility of combining cross-circulation with a rapidly deployable VVB graft as a novel multiorgan biosystemic support approach for liver failure treatment. Utilizing a complete hepatectomy animal model, a magnetic Y-shaped VVB graft enabled rapid bypass deployment, while cross-circulation between the anhepatic and host rats provided physiological support. The results showed that cross-circulation significantly improved biochemical parameters and prolonged survival compared with VVB alone by mimicking essential homeostatic processes that current artificial systems cannot replicate. Although insufficient for long-term rescue in this severe model, the findings contribute important technical innovations with potential applications in staged transplantation, *ex vivo* liver resection, and other novel liver failure therapies. With further optimization in larger animal models, this cross-circulation and VVB graft approach holds promise as an effective strategy to bridge patients to liver transplantation and address critical unmet needs in liver failure management.

## Ethical approval

All animal experiments (No. 2020-602) were performed in accordance with the Animal Welfare Act and approved by the Institutional Animal Care and Use Committee of Xi'an Jiaotong University.

## Consent

This study does not involve patients or volunteers; therefore, ethics committee approval and fully informed written consent were not required and are not documented in the paper.

## Source of funding

This work was supported by the National Natural Science Foundation of China (82000624), Natural Science Basic Research Program of Shaanxi (2024JC-YBMS-653,2022JQ-899 & 2021JM-268), Shaanxi Province Innovation Capability Support Program (2023KJXX-030), Shaanxi Provincial Health and Family Planning Commission Project (2021E018), Shaanxi Province Key R&D Plan University Joint Project-Key Project (2021GXLH-Z-047).

## Author contribution

P.L.: conceptualization, methodology, investigation, and writing – original draft. L.Y.: investigation, methodology, and funding acquisition. Q.L.: methodology. Y.Q.: investigation. A.S.: investigation. X.L.: investigation. S.W.: investigation and funding acquisition. S.F.: investigation and methodology. Y.L.: conceptualization, supervision, and funding acquisition. J.X.: conceptualization, supervision, writing – review and editing, and funding acquisition.

## Conflicts of interest disclosure

The authors declare no conflicts of interest.

## Research registration unique identifying number (UIN)

The current study does not have a Research Registration Unique Identifying Number (UIN).

## Guarantor

Junxi Xiang and Yi Lv.

## Data availability statement

The data are available upon reasonable request.

## Provenance and peer review

Not commissioned, externally peer-reviewed.
